# Cytokine Gene Vaccine Therapy for Treatment of a Brain Tumor

**DOI:** 10.3390/brainsci13111505

**Published:** 2023-10-25

**Authors:** Terry Lichtor, Bingtao Tang, Edward J. Roy

**Affiliations:** 1 Department of Neurological Surgery, Rush University Medical Center, Chicago, IL 60612, USA; 2 Department of Molecular and Integrative Physiology, University of Illinois at Urbana-Champaign, Urbana, IL 61801, USA; bingtaotang@gmail.com (B.T.); eroy@illinois.edu (E.J.R.)

**Keywords:** immunotherapy, IL-2, IL-15, brain tumors, prolonged survival

## Abstract

A glioma is a malignant brain tumor with a poor prognosis. Attempts at the surgical removal of the tumor are the first approach, but additional treatment strategies, including radiation therapy and systemic or local chemotherapy, are necessary. Furthermore, the treatments are often associated with significant adverse side effects. Normal and malignant cells generally have antigenic differences, and this is the rationale for clinical immunotherapeutic strategies. Cytokines such as IL-15 or IL-2, which stimulate an anti-tumor immune response, have been shown to have a particularly high potential for use in immunotherapy against various tumors. In this review, treatments with either a poxvirus, genetically engineered to secrete IL-15, or allogeneic fibroblasts, transfected with tumor DNA and engineered to secrete IL-2, are shown to be effective strategies in extending the survival of mice with malignant brain tumors upon intracerebral injection of the treatment cells. Future studies with these treatment strategies in patients with intracerebral tumors are urgently needed.

## 1. Introduction

### 1.1. Limitation of Current Brain Tumor Treatments

Some increase in the ability to diagnose and surgically treat primary brain tumors has been achieved, although the mortality and overall survival of patients with these tumors has not improved over many years [1]. The present standard treatment modalities, both surgery to remove the tumors and subsequent radiation therapy and chemotherapy, each have significant side effects. The few long-term survivors are inevitably left with cognitive deficits and other disabilities [2,3]. Gliomas are resistant to radiation and standard cytotoxic chemotherapies, making it difficult to treat these tumors [4,5]. Novel therapies are urgently needed.

### 1.2. Principles of Brain Tumor Immunology

Normal and malignant cells generally have antigenic differences, which are the basis for clinical immunotherapeutic strategies. Several different strategies have been attempted to enhance anti-tumor immune responses in mice and patients with intracerebral neoplasms. Immunization with dendritic cells that have been “fed” derivatives of tumor cells or transfected with tumor RNA can result in the development of immune responses against the tumor antigens expressed by malignant cells [6,7]. In patients, immunization with autologous dendritic cells, transfected with mRNA from malignant gliomas, has been found to elicit tumor-specific CD8^+^ cytotoxic T-lymphocyte (CTL) responses against the patient’s malignant cells [8]. Novel and more specific targets, such as glioma stem-like cells, have been shown to increase the success of dendritic cell immunotherapy [9]. Although the results of dendritic cell immunotherapy have demonstrated some good results in animal models, clinical trials have documented few benefits, found to be limited to a minority of treated patients [10].

Another strategy involves the preparation of a vaccine, prepared by the transfer of a cDNA expression library derived from tumor cells into an allogeneic mouse fibroblast cell line expressing a cytokine such as IL-2, which appears to have great potential for the development of an anti-tumor immune response, in the treatment of an intracerebral tumor, in mouse models [11]. Upon the transfer of the cDNA-expression library from the tumor cells into a highly immunogenic fibroblast cell line, genes specifying tumor antigens are expressed. The transferred DNA integrates spontaneously into the genome of the recipient cells and replicates as the cells divide. The transfected fibroblasts can be expanded to obtain quantities for repeated immunizations of the patient. This strategy has the capability of stimulating immunity to a broad array of tumor antigens that characterize the patient’s tumor. Only small amounts of DNA are required to prepare the vaccine, which can be obtained from tumor tissue, such that treatments can be initiated early in the development of the disease. 

In many aggressive tumors, such as gliomas, progression is enhanced by local immunosuppression, associated with an increase in regulatory T cells (Tregs) and myeloid-derived suppressor cells (MDSCs) [12]. The lack of response to treatment in glioma patients may be secondary to the immunosuppressive T cells that normally prevent an anti-tumor immune response [13]. Various cytokines, including interleukin-10 and transforming growth factor-β, have been implicated in the stimulation of Tregs. The targeting of immune checkpoints which regulate the immune system is emerging as a potent and viable cancer therapy [14]. Immunosuppressive mediators, such as IL-10, TGF-β and prostaglandin, can inhibit the function of the immune system and promote the growth of tumors [15]. Reversing the immunosuppressive tumor microenvironment is one of the keys to the success of tumor treatment. 

There are several immunomodulatory cytokines, including IL-2, IL-4, IL-7, IL-9, IL-15 and IL-21, which belong to the family of four α-helix bundle cytokines [16]. The development of IL-2 has been significant in the development of immunotherapy in cancer [17]. However, the use of IL-2 is limited by its toxicity risk and the expansion of regulatory T cells. The associated toxicity issues include hypertension, weight gain, hypothyroidism, cardiac arrhythmias and impaired renal function. To overcome these limitations and improve response rates, other T cell stimulatory agents, such as IL-15, have been in clinical development. IL-15 has been shown to have particularly high potential for use in immunotherapy against various tumors [18]. Furthermore, IL-15, unlike IL-2, does not contribute to the maintenance of regulatory T cells [19]. 

A variety of tumor vaccination strategies have been attempted, including the modification of neoplastic cells to stimulate anti-tumor immune responses. Immunization with tumor cells, modified to secrete immune-augmenting cytokines such as IL-2, IFN-γ and GM-CSF, has resulted in the development of generalized MHC-restricted anti-tumor immune responses in animal models [20,21,22,23,24,25,26,27,28]. Tumor regression has been documented in experimental animals receiving immunotherapy alone [22,23,27,28], which suggests that this treatment strategy may be effective. 

### 1.3. Application of Oncolytic Viruses in Brain Tumor Therapy

Oncolytic virus is a type of virus, either engineered or in nature, which may selectively infect and lyse tumor cells while not affecting normal cells [29]. There are many oncolytic viruses, including herpes simplex virus (HSV), adenovirus, reovirus, poliovirus (PV), vaccinia virus (VV), myxoma virus (vMyx), measles virus, vesicular stomatitis virus (VSV) and Newcastle disease virus [30]. There are multiple potential mechanisms contributing to the selectivity of oncolytic viruses for tumor cells over normal cells. First, viruses can enter tumor cells by binding with certain receptors which are overexpressed on tumor cell surfaces. For instance, HSV binds with herpes virus entry mediators (HVEMs) or nectin-1, VV binds with glycosaminoglycans (GAGs) and VSV binds with low-density lipoprotein receptors (LDLRs) to enter host cells [30]. Second, some of the hyper-activated signaling pathways in tumor cells over normal cells may facilitate virus infection. Hyper-activation of AKT (serine/threonine kinase) is commonly found in most cancer cells, which is a requirement for vMyx infection [31,32]. EGFR activation, common in cancer cells, contributes to a productive infection by the attenuated vaccinia virus JX-594 [33]. Third, the deficiency of tumor cells to Type I interferon responses minimize the anti-viral immune responses, allowing oncolytic viruses to replicate [34,35]. Fourth, the dysfunction of tumor suppressor genes, such as p53, ataxia telangiectasia (ATM) and retinoblastoma protein (Rb), can potentially compromise cellular antiviral activity by accumulating genomic instability and blocking the apoptotic response [36], which contributes to the permissiveness of cancer cells.

Once oncolytic viruses infect tumor cells, they may contribute to the anti-tumor response through a direct cytotoxic effect on tumor cells and the consequent release of tumor-associated antigens, which could stimulate anti-tumor immune responses, turning a “cold” tumor into a “hot” tumor [37]. When the virus is engineered to express an immunostimulatory cytokine [38], it becomes a vector for local expression of potent immune-activating agents, attracting immune cells into the tumor microenvironment (TME) while limiting inflammation.

Many oncolytic viruses have already been tested in several preclinical and clinical trials. T-VEC (also known as Talimogene laherparepvec or OncoVEX^GM-CSF^) was the first oncolytic virus approved for the treatment of advanced melanoma by the U.S. Food and Drug Administration (FDA) in 2015 [39]. T-VEC is an engineered oncolytic herpes simplex virus type 1 (HSV-1), whose neurovirulence factor ICP34.5 is replaced by the gene of human granulocyte–macrophage colony-stimulating factor (hGM-CSF) and whose viral ICP47 gene is deleted [40], to prevent neuronal involvement [41] and enhance anti-tumor efficacy. A OPTiM phase III clinical trial showed great efficacy of T-VEC in targeting patients with early metastatic melanoma (stage IIIB/C-IVM1a) [42]. It also showed enhanced anti-tumor activity when T-VEC was combined with pembrolizumab (anti-programmed death-ligand 1 antibody; PD-1 blockade) in a phase II clinical trial [43]. Furthermore, G47∆, a triple-mutated third-generation oncolytic HSV-1 [44], has a high safety profile and high anti-tumor efficacy (with a 1-year survival rate of 92.3% versus 15%) when targeting human glioblastoma in a phase II clinical trial [45]. 

Poliovirus is another potential candidate for oncolytic virotherapy. The recombinant nonpathogenic polio–rhinovirus chimera (PVSRIPO) is a neuro-attenuated recombinant poliovirus (Sabin vaccine strains), whose internal ribosomal entry site (IRES) was replaced with human rhinovirus type 2 (HRV2) [46]. The result from a phase I clinical trial, where 61 patients with recurrent World Health Organization (WHO) grade IV malignant gliomas were intratumorally infused with PVSRIPO, confirmed the safety of PVSRIPO when used in the brain, and the patients showed a significantly higher survival rate at 24 and 36 months after virus infusion [47]. Studies also showed that PVSRIPO has the potential to have a therapeutic effect on breast cancer, prostate cancer [48] and neuroblastoma [49].

Poxvirus, a group of large, enveloped DNA viruses associated with diseases that generate poxes in the skin, can also be a good choice for oncolytic virotherapy, since the entire replication of poxvirus happens in viral factories within the cytoplasm of infected cells, with no integration of viral DNA into the host genome, which is safe for host cells [50]. Poxviruses can take multiple large foreign genes into their genomes [51] which supports the feasibility of further arming poxviruses (e.g., adding genes of tumor antigen or immune-enhancing cytokines to poxviruses). The vvDD vaccinia virus is a new strain of poxvirus, which was attenuated by the double deletion of the thymidine kinase and the vaccinia growth factor. A preclinical study showed great anti-tumor efficacy when mice bearing MC38 colon cancer or ID8 ovarian cancer were treated with IL15-armed vvDD vaccinia virus. In addition, when combined with a PD-1 blockade, IL15-armed vvDD vaccinia virus led to dramatic tumor regression [52]. It has been reported that IL15-armed myxoma virus (another poxvirus) cured 83% of mice bearing orthotopic gliomas, when combined with adoptive T cell therapy, rapamycin and celecoxib [53].

Despite the promising results, some concerns still need attention when using oncolytic viruses. Safety issues remain a major concern for oncolytic virotherapy. For example, vvDD vaccinia virus, which has undergone two phase I clinical trials and has been found to be safe in humans [54,55], can still be fatal for hosts if it accidentally enters the cerebral lateral ventricle [56]. Therefore, it is essential to study the safety profile of the oncolytic virus in detail before moving to clinical trials. Another concern is the development of the anti-viral immune responses mediated by neutralizing antibodies [57] and immune cells, such as macrophages [58] and natural killer [59] (NK) cells. 

## 2. Pre-Clinical Experimental Findings

### 2.1. Survival of Mice with Intracerebral Gliomas upon Treatment with Fibroblasts Engineered to Secrete Cytokines

Gl261 cells are a glioma cell-line of C57Bl/6 mouse origin (H-2^b^). LM fibroblasts are derived from C3H/He mice and express H-2^k^ determinants. The potential development of an anti-tumor immune response was explored, using fibroblasts engineered to secrete either IL-2 or IL-2 and interferon-γ, injected into the brains of mice with an intracerebral (i.c.) glioma [60]. Glioma cells were mixed with cells secreting one or two of the cytokines of interest and subsequently injected into the right frontal lobe of C57BL/6 mice, which are syngeneic with G1261 cells. The results demonstrate that IL-2-secreting fibroblasts were capable of prolonging survival in mice with a right frontal glioma upon i.c. injection of the treatment cells (*p* < 0.025). The results were more dramatic upon i.c. injection of mice with a glioma treated with LM-IL-2/interferon-γ double cytokine-secreting cells (*p* < 0.005). The i.c. injection of mice with equivalent numbers of LM-IL-2 cells without a tumor lived many months and did not demonstrate either ill effects or a neurologic deficit.

### 2.2. Immunocytotoxic Studies from Spleen Cells of Mice Treated with Allogeneic Cytokine Secreting Fibroblasts

A chromium release assay was used to determine the reactivity of spleen cells from the immunized mice to chromium-labeled Gl261 glioma cells. The results [60] demonstrate a significantly elevated chromium release when spleen cells, from the mice with i.c. Gl261 cells treated in the brain with IL-2 secreting fibroblasts, were co-incubated with chromium-labeled Gl261 cells (*p* < 0.05). This demonstrates that an anti-tumor immune response developed systemically in the mice with gliomas, following the intracerebral injection of cells secreting either IL-2 or IL-2/IFN-γ. Antibody depletion studies reveal that CD8^+^ and NK/LAK cells were responsible for the anti-tumor immunity.

### 2.3. Treatment of Gliomas in Mice Treated with IL-15 Secreting Cells

Two oncolytic poxviruses, the vvDD vaccina virus and the myxoma virus, were engineered to express the fusion protein IL15Ra-IL15 [53]. Viral gene expression was confirmed in the murine glioma by staining for M-T7 (a myxoma-encoded protein) and IL-15. Mice with a glioma were treated with either of these two viruses engineered to secrete IL-15 [61] supplemented by rapamycin, celecoxib and adoptive T cell therapy (using tumor-specific CD8^+^ T cells). Rapamycin was used to enhance the spread and replication of oncolytic viruses [62,63,64], while celecoxib was intended to reduce the immunosuppressive tumor microenvironment by inhibiting the production of prostaglandins (mainly PGE2) [15,65]. The rationale for this treatment is that the oncolytic poxvirus could lead to a cytotoxic effect on glioma cells, followed by release of potential tumor antigens, which may generate a stronger anti-tumor immune response. Direct injection of vvDD-IL15-Rα into the lateral cerebral ventricles was uniformly fatal, whereas mice that received intracerebral vMyx-IL15Rα-tdTr injections recovered from the virus infection [56]. This suggests that vvDD vaccinia virus may not be a safe choice to treat tumors inside of the brain, whereas myxoma virus could be a potential alternative. Mice that received intracerebral vMyx-IL15Rα-tdTr injection recovered from the virus infection [56].

**Figure 1:**
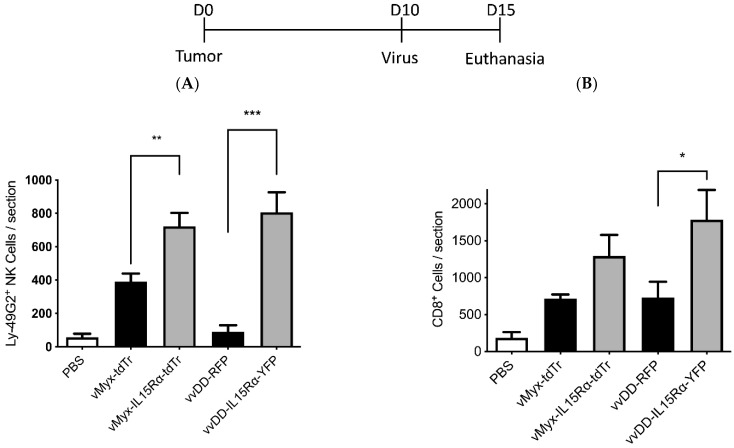
Presence of NK and CD8+ cells in tumor sections of mice in various treatment groups. C57BL/6J mice were implanted with GL261 NS cells i.c., followed 10 days later by injection with PBS, vMyx-tdTr, vMyx-IL15Rα-tdTr, vvDD-CXCL11 or vvDD-IL15Rα-YFP and PBS, vMyx-tdTr, vMyx-IL15Rα-tdTr, vvDD-RFP or vvDD-IL15Rα-YFP; (n = 3) (2 × 10^6^ pfu intratumoral). vMyx-tdTr is the myxoma virus carrying the gene of tdTomato Red; vMyx-IL15Rα-tdTr is the myxoma virus carrying the genes of IL15Ra-IL15 and tdTomato Red. vvDD-RFP is the vvDD vaccinia virus carrying the gene of RFP (red fluorescent protein); vvDD-IL15Rα-YFP is the vvDD vaccinia virus carrying the genes of IL15Ra-IL15 and YFP (yellow fluorescent protein). Mice were euthanized 5 days after the virus treatment and tumor sections were analyzed for NK cells and CD8^+^ T cells by immunostaining. (**A**) Number of Ly-49G2^+^ NK cells per tumor section for each condition; mean values and SEM are shown. One-way ANOVA showed significant increase in NK cell accumulation in vMyx-IL15Rα-tdTr- or vvDD-IL15Rα-YFP-treated tumors, compared to vMyx-tdTr or vvDD-RFP treatments (** *p* ˂ 0.01; *** *p* ˂ 0.001). (**B**) Number of CD8^+^ cells per tumor section for each condition; mean values and SEM are shown. One-way ANOVA showed significant increase in CD8^+^ cell accumulation in vvDD-IL15Rα-YFP-treated tumors compared to vvDD-RFP treatment (* *p* ˂ 0.05). The work for this study was carried out in the laboratory of co-authors Dr. Bingtao Tang and Dr. Edward Roy.

To explore the anti-tumor efficacy of myxoma virus expressing IL15, the combination treatment (vMyx-IL15Rα-tdTr, rapamycin, celecoxib, and adoptive T cell transfer therapy) was applied to mice with gliomas. An increased number of infiltrating NK and CD8^+^ T cells was detected in the tumor specimens (Figure 1A,B) indicating that the IL15Rα-IL15 fusion protein is biologically functional and could attract NK and CD8^+^ T cells into the tumor site. The prolongation of survival (*p* < 0.05, compared to untreated animals or animals that did not receive celecoxib) indicated that myxoma virus (vMyx-IL15Rα-tdTr), supplemented by rapamycin and the prostaglandin inhibitor celecoxib, could provide a safe and effective anti-tumor treatment in mice with intracerebral gliomas, upon the injection of treatment cells into the brain or lateral ventricles. The efficacy of this novel combination treatment strategy in glioma-bearing mice, following tumor resection, is being explored [66].

### 2.4. Immunization with Allogeneic Cytokine-Secreting Fibroblasts Transfected with DNA from Breast Cancer in Treatment of C3H Mice with Intracerebral Breast Cancer

A tumor vaccine was constructed by the transfer of DNA from a breast cancer cell line (SB-5b) that arose in C3H mice (H-2^K^) into cytokine-secreting mouse fibroblasts (H-2^K^). The application of these cells as a potential treatment of an intracerebral breast neoplasm arising in C3H mice was investigated. The cells were modified to express H-2K^b^ determinants, and this was intended to also ensure rejection. Studies with these cells revealed that there was a prolongation of survival in C3H mice with intracerebral breast cancer, upon treatment by immunization with fibroblasts secreting either IL-2 or GM-CSF transfected with DNA from the same spontaneous breast neoplasm (*p* < 0.05) [67].

### 2.5. Splenic T Cells Reactive with SB-5b Tumor Cells in Mice Immunized with Transfected Cytokine Secreting Fibroblasts

An ELISPOT-IFN-γ assay was used to estimate the development of splenic T cells, reactive with SB-5b cells, in mice immunized with transfected fibroblasts modified to secrete IL-2 or GM-CSF. The assay was performed six weeks after the i.c. injection of the mixture of SB-5b cells and the transfected fibroblasts. The findings in these studies revealed that the highest proportion of T cells reactive with SB-5b cells was in surviving mice injected with fibroblasts modified to secrete IL-2. Lesser numbers of spots were found in T cells from mice injected with SB-5b cells and non-secreting transfected fibroblasts or SB-5b cells and transfected fibroblasts modified to secrete GM-CSF.

In additional experiments, animals with i.c. breast cancer were treated i.c. with LMK^b^IL-2/SB5b cells. An ELISPOT assay was undertaken after two weeks, using the spleen cells to detect IFN-γ secretion in the presence or absence of SB-5b tumor cells and antibodies against various T cell subsets. These studies revealed that CD4^+^, CD8^+^ and NK/LAK cells were responsible for the anti-tumor immune response. The overall *p*-value between the unstimulated and the tumor cell stimulated group was *p* < 0.001.

### 2.6. Development of an Enrichment Strategy for a More Potent Vaccine

A strategy was developed to enrich the vaccine for efficacy by identifying cell populations that were the most highly immunogenic [11]. Populations with higher numbers of immunogenic cytokine secreting cells transfected with tumor DNA were identified by their stronger anti-tumor immune response against SB5b cells in C3H/He mice. Two sub pools that stimulated immunity to the greatest (immuno^high^ pool) and least (immuno^low^ pool) after three rounds of enrichment were identified and used in further studies.

### 2.7. Development of Systemic Anti-Tumor Immunity in the Treated Animals

**Figure 2:**
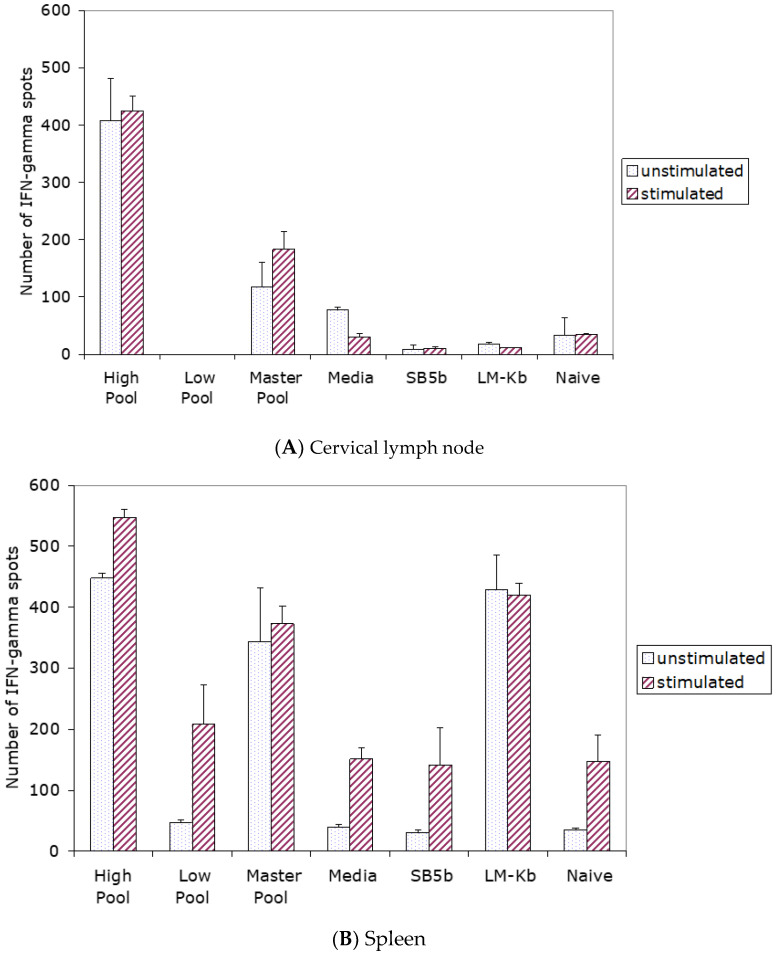
Increased numbers of responding T cells were detected in the spleens and cervical lymph nodes of naïve mice, which were injected i.c. with cells from the various treatment groups. To determine if systemic anti-tumor immunity was generated in tumor-free mice injected i.c. with cells from the immuno^high^ pool, cervical lymph node and spleen cells from the injected mice were analyzed by ELISPOT IFN-γ assays for responding T cells. Naïve C3H/He mice received 2 i.c. injections at weekly intervals of 1.0 × 10^6^ cells from the immuno^high^ pool. One week after the second injection, mononuclear cells from the spleens and cervical lymph nodes of the immunized mice were analyzed for the presence of T cells responsive to the breast cancer cells. For the controls, an equivalent number of cells from the non-selected master pool, or cells from the immuno^low^ pool, were substituted for cells from the immuno^high^ pool. For the additional controls, the same protocol was followed except that the mice were injected i.c. with equivalent numbers of SB5b cells, with LMK^b^ cells or with media. Mice injected with SB5b tumor cells received only one injection. Mononuclear cells from the spleens of the mice were co-incubated with (stimulated) or without (unstimulated) SB5b cells. The results illustrated in this figure indicate that the highest number of responding cervical lymph node (**A**) or spleen cells (**B**) were in mice injected i.c. with cells from the immuno^high^ pool (*p* < 0.05 vs. the number of responding spleen cells in mice injected with cells from the master pool, and *p* < 0.005 vs. cells from mice in any of the other groups from the cervical lymph nodes). The work for this study was undertaken in the laboratory of Terry Lichtor, MD, PhD, who is one of the authors of this paper.

To analyze the development of systemic anti-tumor immunity in tumor-free mice injected i.c. with cells from the various treatment groups, ELISPOT IFN-γ assays for the measurement of responding T cells were carried out using cells from the cervical lymph nodes and spleens from the injected mice. A micro cannula was placed into the right frontal lobe of naïve C3H/He mice. Following the cannula insertion, the animals were injected into the brain through the cannula with 1.0 × 10^6^ cells from the immuno^high^ pool on two days separated by one week. For the controls, the same procedure was followed, except that the cells from the non-enriched master pool or cells from the immuno^low^ pool were substituted for cells from the immuno^high^ pool. For the additional controls, the tumor-bearing mice were injected into the brain with equivalent numbers of non-DNA-transfected LMK^b^ cells or with SB5b tumor cells alone. Mice injected with SB5b tumor cells received only one injection. The data revealed that the highest number of responding T cells were in the cervical lymph nodes (Figure 2A) or spleens (Figure 2B) of mice injected i.c. with cells from the immuno^high^ pool (*p* < 0.05).

### 2.8. Stimulation of T Cell Subsets in the Spleens of Mice with i.c. Breast Cancer following Treatment of the Tumor with Cells from the Immuno^high^ Pool

The effect of antibodies against various T cell subsets on the responding T cell response was used to determine the types of cells activated for anti-tumor immunity in the spleens of mice treated with cells from the immuno^high^ pool, using the above protocol. ELISPOT IFN-γ assays were used for this analysis. The anti-tumor immune response was inhibited to the greatest extent by antibodies against CD4^+^ cells. The results were less dramatic if the spleen cells were incubated in the media containing CD8^+^ or NK/LAK antibodies.

### 2.9. Decreased Number of T-Reg Cells in the Spleens of Mice with i.c. Breast Cancer Treated with Cells from the Immuno^high^ Pool

T-reg cells are potent inhibitors of natural anti-tumor immunity [68]. Protocols involving immunotherapy strategies are affected to a certain degree by the numbers of T-reg cells and cytotoxic T lymphocytes in tumor-bearing animals and patients, due to their inhibition of the immune system. Quantitative RT-PCR for Foxp3, a transcription factor characteristic of T-reg cells, was used to measure the number of T-reg cells in the spleens and brains of mice with i.c. breast cancer treated by i.c. injection of cells from the immuno^high^ pool. The findings from these studies reveal that Foxp3^+^ T-reg cells were relatively deficient in the spleens from the animals with i.c. breast cancer treated with cells the immuno^high^ pool. An analysis of the spleen cells by FACS revealed that mice injected i.c. with cells from the immuno^high^ pool had a relative deficiency of CD4^+^/CD25^+^/Foxp3^+^ T cells, and a corresponding increase in the numbers of CD8^+^ cells.

### 2.10. Prolonged Survival of Mice with i.c. Breast Cancer Treated by Injection into the Tumor Bed of Cells from the Immuno^high^ Pool

To explore the potential prolongation of survival in mice with i.c. breast cancer treated by cells from the immuno^high^ pool, C3H/He mice were injected i.c. with 5.0 × 10^4^ SB5b cells and 1.0 × 10^6^ cells from the immuno^high^ pool. The findings from these studies revealed that mice with i.c. breast cancer treated with cells from the immuno^high^ pool survived significantly longer than untreated mice (*p* < 0.05); the treated animals survived 26 days while the untreated animals survived only 14 days. Similar results were demonstrated if the mice with i.c. breast cancer cells were treated with the subcutaneous (s.c.) injection of cells from the immuno^high^ pool.

## 3. Future Perspectives for Tumor Vaccines

The heterogenicity and weakly immunogenic composition of gliomas makes these tumors difficult to treat. A limited number of phase III immunotherapy trials have been completed for checkpoint inhibitors, monoclonal antibodies and dendritic cell vaccines, which have not demonstrated an improvement in survival outcomes [69,70]. One of the most promising anti-tumor immunotherapy strategies, involving the use of dendritic cells pulsed with known peptides, has been explored in a large clinical trial, but no phase III clinical trial has achieved a positive endpoint [71]. SurvaxM is a peptide vaccine conjugate that has been shown to activate the immune system against its target molecule survivin, which is highly expressed by glioblastoma cells; a large randomized clinical trial for SurvaxM is in progress [72]. Alphaviruses have also been recently developed for immunotherapy involving several tumors, including gliomas. Immunization with this virus has demonstrated reduced tumor growth associated with some therapeutic efficacy, and vaccinated animals have shown protection against challenges with tumor cells. A limited number of phase I clinical trials using this virus have been developed for patients with a variety of tumors [73].

## 4. Conclusions

In summary, the data from this study suggest that a poxvirus genetically engineered to secrete IL-15, along with a prostaglandin synthesis inhibitor to block immunosuppression, is an effective treatment for increasing survival in mice with a malignant glioma. Two different oncolytic poxviruses expressing the IL15Rα-IL15 fusion protein were studied. Cytokines such as IL-2 and IL-15 are beginning to find a role in the management of patients with malignant gliomas [74,75]. Early results, however, have not revealed significant efficacy for prolonging survival in patients with brain tumors through the use various cytokines, including IL-2 and IL-15 [76].

Oncolytic viruses may contribute to the anti-tumor response through direct cytotoxic effects on cancer cells, such that there is a release of tumor antigens which may stimulate an immune response. Previous studies have shown that the oncolytic poxvirus, myxoma virus, is safe to use in mice even when directly injected into the cerebral ventricles [77]. When the virus is engineered to express a cytokine such as IL-15, it can stimulate the local expression of potent immune-activating agents, while avoiding systemic inflammation that parenteral delivery of the cytokine would produce. In this study, IL15 was chosen because it activates and maintains the function of NK and CD8^+^ T cells [61] with less potential for vascular leakage [78] and less activation of Tregs [79].

In other studies, it was shown that a vaccine prepared by the transfer of a cDNA expression library, derived from tumor cells, into a mouse fibroblast cell line engineered to secrete IL-2 appears to be efficacious in the treatment of a brain tumor. The cDNA integrates spontaneously into the genome of the recipient cells, which is followed by replication and expression. The vaccine requires only a small amount of tumor tissue for preparation and can be given at an early stage of the disease, when the tumor is most susceptible to immune-based therapy. A unique enrichment strategy was also developed, such that the proportion of active immunotherapeutic cells in the vaccine is increased.

The use of cells from the enriched vaccine was associated with the development of a strong anti-tumor immune response. The predominant cell type activated in mice immunized with cells from the immuno^high^ pool were CD4^+^ T cells. The treatment of a brain tumor with cells from the immuno^high^ pool also resulted in a reduction in the relative numbers of T-reg cells, with a potential prevention of the impaired anti-tumor immune responses frequently found in patients with malignant brain tumors.

The goal of tumor treatment would be the removal of every tumor cell. A single therapy will likely not achieve this goal in the case of brain tumors. However, immunotherapy, in combination with surgical removal of the tumor, radiation therapy and chemotherapy, will likely be involved in the management of patients with brain tumors. The development of DNA-based tumor vaccines, in combination with cytokine secretion, is a novel strategy that does not require antigen identification or protein purification, and yet can elicit a potent and long-lasting activation of the immune response, which will lead to the rejection of the tumor. Only small quantities of tumor-derived DNA, from surgical specimens, are needed to prepare the vaccine. The enrichment strategy represents a unique approach to isolating highly immunogenic pools of transfected cells, which leads to the development of enhanced anti-tumor immunity. Thus DNA-based cytokine-secreting vaccines offer several unique advantages, which support their further development as an immunotherapeutic treatment option for patients with a variety of tumors in general and, in particular, patients with malignant intracerebral tumors. The use of viruses engineered to secrete cytokines remains another treatment option and requires further study regarding the management of these tumors.
